# Schistosome Transgenesis: The Long Road to Success

**DOI:** 10.3390/biology13010048

**Published:** 2024-01-16

**Authors:** Bernd H. Kalinna, Allen G. Ross, Anna K. Walduck

**Affiliations:** Rural Health Research Institute, Charles Sturt University, Orange, NSW 2800, Australia; agpross@csu.edu.au (A.G.R.); anwalduck@csu.edu.au (A.K.W.)

**Keywords:** schistosomiasis, transfection, functional genomics, mobile genetic elements, RNA interference, RNAi, genome editing, CRISPR, genomic safe harbour site

## Abstract

**Simple Summary:**

The neglected tropical disease schistosomiasis is a worm infection that is caused by parasitic blood flukes. The disease is found in sub-Saharan Africa, the Middle East, Southeast Asia, and the New World. Worldwide, 240 million people are infected, and 700 million people are at risk. Schistosomiasis is a debilitating, chronic disease, and the mortality is estimated at 200,000 deaths per year. Schistosomiasis control relies on the drug praziquantel, but it does not prevent reinfection after treatment. The development of new vaccines, drugs, and diagnostic methods and the investigation of the biological basis of infectivity are, therefore, of critical importance. The development of transgenesis systems, as have been used for other pathogens, has been hampered by the complexity of the parasite and its life cycle. Now, after 25 years, tools to genetically manipulate the parasite have developed to the point that we can analyse this parasite’s biology in detail. In this review, we describe the progress that has been made in establishing gene manipulation in schistosomes and discuss numerous examples of studies that have led to a better understanding of molecules that are essential for host–parasite interplay. This insight will lead to new strategies and methods for combatting and managing schistosomiasis.

**Abstract:**

As research on parasitic helminths has entered the post-genomic era, research efforts have turned to deciphering the function of genes in the public databases of genome sequences. It is hoped that, by understanding the role of parasite genes in maintaining their parasitic lifestyle, critical insights can be gained to develop new intervention and control strategies. Methods to manipulate and transform parasitic worms are now developed to a point where it has become possible to gain a comprehensive understanding of the molecular mechanisms underlying host–parasite interplay, and here, we summarise and discuss the advances that have been made in schistosome transgenesis over the past 25 years. The ability to genetically manipulate schistosomes holds promise in finding new ways to control schistosomiasis, which ultimately may lead to the eradication of this debilitating disease.

## 1. Introduction

Schistosomes belong to the phylum Platyhelminthes (flatworms) and the class Trematoda (flukes). They are digenetic blood trematodes and cause schistosomiasis in humans and other mammals by depositing eggs in the circulatory system surrounding the gut or bladder of the infected host. Five species of schistosomes have medical importance, and they can be divided into urinary schistosomes (*Schistosoma haematobium*) and intestinal schistosomes (*S. mansoni*, *S. japonicum*, *S. mekongi*, and *S. intercalatum*). This distinction is based on the pathology they cause, egg morphology, and the specific snail intermediate hosts that play an essential role in transmission.

Geographically, schistosomes are found in sub-Saharan Africa; the Middle East; Southeast Asia; and the New World in Brazil, Saint Lucia, Suriname, Venezuela, and the Caribbeans. Schistosomiasis is a neglected tropical disease with over 240 million cases worldwide, and an estimated 700 million people are at risk of infection. The disease is responsible for the loss of over 2.5 million disability-adjusted life years (DALYs), making it the third-highest global burden caused by a neglected tropical disease. The mortality of schistosomiasis is estimated at 200,000 deaths annually [1], and the morbidity (e.g., anaemia, cognitive impairment, malnutrition, and growth stunting) is in the range of 1.7–4.5 million disability-adjusted life years (DALYs) per annum [2], which is a testament to the public health significance of this disease. Schistosomiasis has been targeted by the WHO for elimination as a public health problem by 2030 [2].

**Figure 1:**
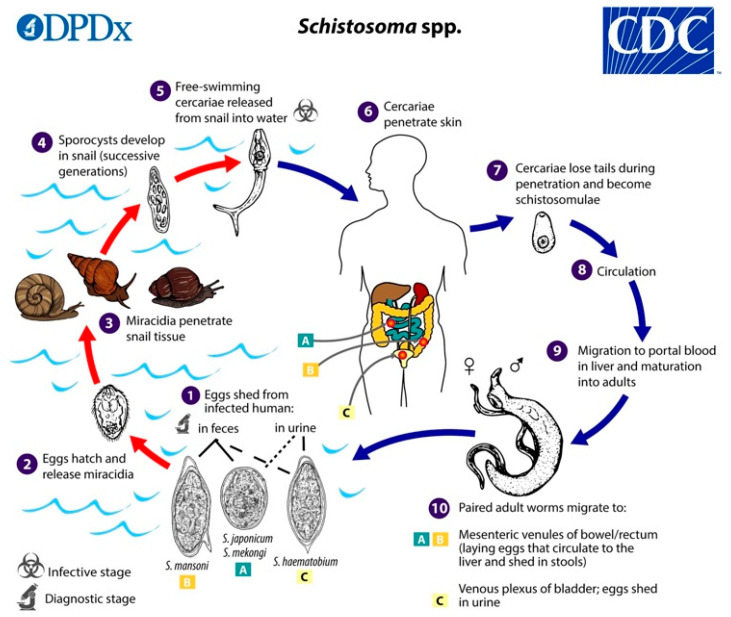
Life cycle of *Schistosoma* spp. *Schistosome* eggs are shed into fresh water from infected humans with faeces or urine, depending on species (1): 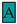 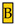 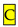. The first larval stages of the parasite (miracidia) hatch from eggs (2) and seek out and penetrate specific snail intermediate hosts (3). In the snail, the miracidia develop into two consecutive generations of sporocysts (4), which produce large numbers of cercariae, the second larval form of the parasite (5). Cercariae are released from the snail and seek out and penetrate the skin of the human host (6). During penetration. they shed their forked tails to become schistosomulae (7). These juvenile worms migrate via the venous circulation to the lung and heart and finally develop in the liver (8) and (9). Male and female adult worms copulate and finally reside in the mesenteric venules of the bowel/rectum or the venous plexus of the bladder (10) (see 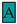, 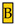, and 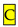 for specific locations depending on schistosome species) [10].

The schistosome life cycle (Figure 1) is complex and involves a sexual life-cycle phase in adult worms that reside in the definitive host and two asexual reproductive phases in the larval stages that occur in the intermediate snail host with the involvement of eggs, miracidia, mother sporocysts, daughter sporocysts, and cercariae. This asexual phase considerably increases the number of infective larvae that are released into the environment. Transmission occurs in contaminated fresh water through contact between the definitive host (e.g., humans) and the free-living larval-stage cercariae. Infected humans or bovines release eggs into the environment, which release miracidia, which later infect the snail intermediate host to complete the life cycle of the parasite. Although the adult worms reside in the circulation, the major cause of pathology is eggs of the parasite that, in the case of intestinal schistosomes, are deposited in the intestinal wall and the liver. In urinary schistosomiasis, eggs become lodged in the bladder wall, which leads to blood in the urine and inflammation.

If untreated, the disease can become chronic, with severe outcomes such as accumulation of fluid in the peritoneal cavity causing abdominal swelling; hepatosplenomegaly; portal hypertension; liver fibrosis; and, in the case of urinary schistosomiasis, secondary cancers of the bladder and death. In 5–6% of patients, the parasite may enter the central nervous system and cause further complications. The manifestations of chronic schistosomiasis are a result of a long-term battle between the host immune system and parasite evasion mechanisms. Broadly, decades of research have revealed that initial exposure triggers a Th1-biased inflammatory response, but as the parasite matures, egg secretions drive a Th2-biased, regulatory, and granulomatous response that affects liver function (see [3,4] for excellent reviews). The inflammatory milieu generated by chronic schistosomiasis also impacts the outcome of other infections present in endemic areas, and co-infected individuals have been shown to have altered antibody responses to *P. falciparum* malaria [5] and *Helicobacter pylori* [6], for example. The impact of infection extends to vaccination, and *S. mansoni*-infected individuals have reduced responses to a Hepatitis B vaccine [7]. In addition, bystander effects on non-infectious conditions such as colitis [8] and asthma [9] have also been reported in animal models.

## 2. Limitations of Current Control Strategies

Praziquantel has been the basis of schistosomiasis control since its discovery in the 1970s. The drug is highly effective in killing adult worms of all schistosome species that infect human subjects, and when given to people with schistosomiasis, it controls morbidity well [11,12]. However, praziquantel does not kill immature schistosomes and cannot prevent reinfection. Praziquantel-based control programs have, therefore, only a temporary effect on transmission and, in addition, have limited potential to interrupt disease transmission in the long term [11,12,13]. In endemic areas, once mass treatment with praziquantel is stopped, disease prevalence can return to baseline levels within 18–24 months [14,15]. 

For many bacterial and viral infectious diseases, vaccination has been shown to be one of the best control options. This is in stark contrast to the availability of vaccines for diseases caused by parasitic helminths, including those caused by schistosomes. Despite a decade-spanning effort, the development of a protective and therapeutic vaccine against schistosomiasis has been exceptionally difficult. This is likely due to the complex life cycle of blood flukes, where the immune system of patients is confronted with several life-cycle stages of the parasite: cercariae, schistosomula, adult worms, and eggs. Research has shown that each life-cycle stage expresses numerous antigenic molecules with the potential to elicit strong humoral and cellular immune responses [16]. In chronic schistosome infections, the antigen-specific responses to some antigens are upregulated, while the responses to others are, at the same time, downregulated. This phenomenon can most likely be attributed to the switch from a Th1-biased inflammatory response to a Th2-dominated regulatory response [3,4]. Generally, resistance to infection seems to be entirely dependent on antibody responses and a strong association with interferon-g and interleukin-12 production [17]. Another intriguing aspect of the development of a protective immune response has emerged from epidemiological studies of people living in endemic areas. Here, it has been shown that children are more susceptible to infection and reinfection after praziquantel treatment compared with the adult population [18]. A hypothesis has been put forward stating that antigens rereleased from dead worms cross-react with antigens from fresh migrating larvae, thereby stimulating a protective IgE response. As a consequence, natural immunity might only develop if exposure is maintained over a long time and after many infection cycles [19]. 

With the advance of genomics and proteomics in the early 2000s, there was hope that investigating the biology of schistosomes on a molecular level could contribute to a better understanding of the relationship between hosts and parasites to underpin the development of new vaccines, drugs, and diagnostic methods and to investigate the biological basis of infectivity, pathology, antigenicity, and drug resistance. To this end, transgenesis tools and, in particular, techniques to systematically inactivate gene function have been essential for investigating gene function in other organisms (e.g., bacteria, yeast, Drosophila, *C. elegans*, and zebrafish) [20,21,22]. There is a need to develop similar tools for schistosomes to enable somatic and germline transgenesis, which would enable the validation of genes or gene products as potential targets for drug or vaccine development. The genome sequences of *S. mansoni* and *S. japonicum* were published in 2009 [23], and since then, numerous approaches and techniques have been established and utilised to enable forward and reverse genetics in this parasite. 

In the following, we present a historic overview of the advances in schistosome transgenesis, followed by a discussion of recent developments that have led to the first successes in better understanding the complex biology of schistosomes. 

## 3. Transient Transfection of Schistosomes

### 3.1. Biolistics

**Figure 2:**
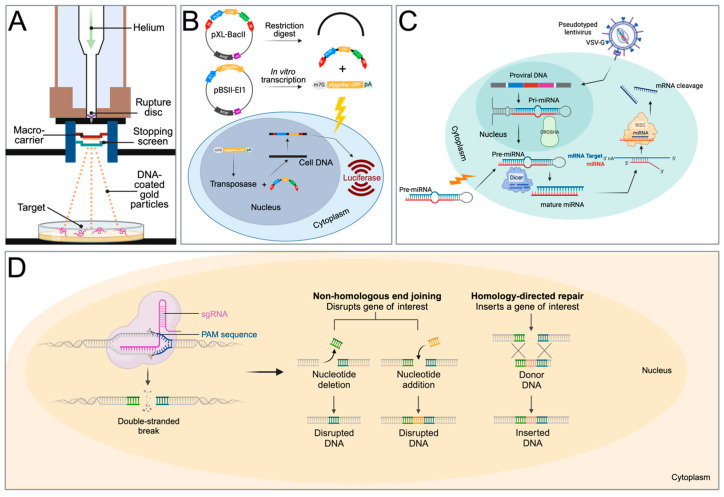
Transfection methods used for transgenesis in schistosomes. (**A**) Particle bombardment (gene gun) for the transfer of expression plasmid-covered gold particles. (**B**) Two-component *piggyBac* transposon system. pXL-BacII includes the *piggyBac* terminal inverted repeats (red arrows), an ampicillin resistance gene, a bacterial ColE1 replication origin, and the *S. mansoni* actin 1.1 gene promoter, driving the firefly luciferase gene. pBSII-IE1-orf is a *piggyBac* helper plasmid, providing the *piggyBac* transposase under the control of the IE1 promotor. After a restriction digest of pXL-BacII and *in vitro* transcription of the transposase from pBSII-IE1-orf, the IR-Act-Luc-IR cassette and transposase mRNA are transferred into schistosomes via electroporation, leading to luciferase expression. (**C**) Gene suppression by micro-RNA. The precursor RNA is either delivered into the cell via electroporation or with a lentiviral construct. In lentiviral delivery, DROSHA ribonuclease III cleaves primary miRNA (pri-miRNA) hairpins in the nucleus. The resulting precursor miRNA (pre-miRNA) is exported to the cytoplasm. The pre-miRNA is further processed by DICER to produce mature miRNA duplexes. RISC uses the miRNA as a template for recognising complementary mRNA. When a complementary strand is found, it activates RNase and cleaves the RNA. VSV-G: Vesicular stomatitis virus glycoprotein. (**D**) Gene editing with CRISPR. After the delivery of the Cas9 protein and sgRNA via electroporation, the target DNA is cut. Non-homologous end-joining leads to a disruption of the targeted gene (knockout), while homology-directed repair leads to gene knock-in with the help of a homology repair donor DNA. Created with BioRender.com.

The pioneering reports that established that transgenesis is, in principle, possible for schistosomes employed biolistic particle bombardment (gene gun) to transfer plasmids that encode reporter genes, such as jellyfish green fluorescent protein (GFP) or firefly luciferase, into schistosomes (Figure 2A). Davis et al. (1999) were the first to show that the biolistic transfection of adult schistosomes with a plasmid containing the luciferase gene driven by the *S. mansoni* Splice Leader RNA gene promotor or the bombardment of the parasites with a m^7^G-capped, polyadenylated luciferase mRNA resulted in ~20-fold-higher expression levels in the luciferase reporter 11–40 h after transfection when compared with untreated controls [24]. Despite this success in showing that foreign genes are expressed in schistosomes, the report did not provide microscopy data to show the location of the gene expression, nor did it specify whether the worms were damaged by the procedure. After this initial report, several studies were published to address these questions by employing GFP for ease of transgene detection, extending the application of biolistic transformation to investigate schistosome protease genes [25,26,27,28,29].

First, Wippersteg et al. (2002) used a plasmid consisting of the heat shock protein 70 (*hsp70*) gene promoter and terminator sequences of *S. mansoni* in fusion with the GFP reporter gene. After the transfection of adult worms and sporocysts, GFP expression was evident on the surface of adult worms and in the internal structures of the sporocyst. By using reverse transcription PCR and Western Blot, the authors confirmed that the transgene was transcribed and translated [25].

The investigations were then extended to characterise the *S. mansoni* ER60 cysteine protease [26,27], which is associated with the excretory system (ES) of these parasites. The promoter and terminator regions of the *ER60* gene were fused with the GFP sequence to investigate the expression pattern of ER60–green fluorescent protein in sporocysts after transfection via particle bombardment. The results were consistent with the expected expression in the excretory system, with tissue-specific GFP fluorescence visible in the gland cells, protonephridia, and cytons of the larvae. This was further corroborated by co-localisation with Texas Red-BSA, which has the ability to specifically enter the excretory system of living schistosomes. The results suggested that the ER60 protease is expressed in the ES of the larvae and possibly plays a role in penetration and migration in the definitive host.

Similarly, additional schistosome proteins have been investigated using biolistic transfection [28,29,30]. Here, 5′ flanking fragments of the cathepsin L (*SmCL*), D (*SmCD*), F (*SmCF*), and B2 (*SmCB2*) peptidase genes and the calcineurin A gene (*SmCNA*) were used to construct GFP expression vectors, which were then utilised to transfect schistosome worms. The observed expression patterns of SmCL; SmCD in the gut [28]; SmCF and SmCB2 in the gut and tegument, respectively [29]; and SmCNA in the excretory system [30] was confirmed by the tissue-specific localisation of the EGFP reporter after particle bombardment.

Taken together, the studies discussed above firmly established that schistosomes are amenable to transfection and that valuable insights into the biology of this parasite could be gained using particle bombardment as a means of transfection. The main disadvantage of this method, however, is the transient nature of the transgene expression, the inability to target specific tissues of the parasite, and the difficulty in achieving germline transfection. To address this problem, our group used particle bombardment to target the germline in miracidia, the larval form of the parasite that infects the intermediate snail host and gives rise to successive generations of sporocysts and, ultimately, cercariae that infect the definitive host. We were able to show that a plasmid that encodes an enhanced green fluorescent protein (EGFP) under the control of the *S. mansoni HSP70* promoter and termination elements was able to drive the expression of the reporter gene in miracidia. Transfected miracidia were able to penetrate and establish, in *Biomphalaria glabrata*, the *S. mansoni* intermediate host snail, and gold particles could be detected in the germ balls of the parasites in paraffin sections of snail tissue. In addition, the miracidia transformed into mother sporocysts inside the snails, and reporter gene activity could be determined at 10 days post-infection using RT-PCR on snail tissues [31]. This was the first study to show that it is feasible to return transgenic miracidia to the parasite’s life cycle, a crucial step in the establishment of transgenesis in schistosomes. Our study was extended by Beckman et al. (2007), and in their work, after the bombardment of miracidia, the transgene was also detectable in cercariae and adults of the F0 and F1 generations [32]. 

### 3.2. Transposable Elements

At the same time that particle bombardment was being explored as a means of transfecting schistosomes, a different concept was gaining interest. After the release of the schistosome genome [33,34], it became apparent that these parasites have unusually large genomes. The nuclear genome comprises 363 megabases containing ~12,000 genes, which are distributed over seven pairs of autosomes and one pair of sex chromosomes, and account for about 50% of the genome [35,36]. The remaining 50% is composed of repetitive sequences, with many related to transposable elements [37].

Transposable elements (TEs), or mobile genetic elements, are DNA sequences that replicate independently in the genome. TEs consist of three major groups, with Class I representing retrotransposons, which spread through the genome via a copy/paste mechanism, creating multiple repetitive sequences throughout the genome. Class II TEs also move through the genome through a cut/paste mechanism; they are, however, not copied but rather move through the genome employing a transposase encoded by the Class II TE itself, which facilitates the cutting and pasting process. Class III TEs, also called miniature inverted-repeat transposable elements (MITEs), are relatively short elements and contain inverted repeats at each end. As most can be found in euchromatin, it has been suggested that they might be involved in regulatory functions. A comprehensive overview of eukaryotic transposable elements can be found elsewhere [38].

In schistosomes, both Class I (*Boudicca*, *Sinbad*, *Gulliver*, *Saci-1*, *Saci-2*, *Saci-3*, *Fugitive*, and *Perere*) [39,40,41] and Class II (*Merlin*, *SmTRC1*) [42,43] elements have been identified and further characterised. In bacterial artificial chromosomes prepared from *S. mansoni* genomic DNA, we detected three open reading frames (ORFs) that were bounded by long terminal repeats (LTRs). The ORFs encoded sequences with homology to Group Antigen (GAG) polyproteins, reverse transcriptase, RNaseH, integrase, and a putative envelope protein, domains that are also found in retrotransposons and retroviruses [44]. The order of these domains in the genome of schistosomes was similar to that found in the gypsy/Ty3 retrotransposons, which are widely distributed among animals, plants, and fungi [45]. Analysis of the reverse transcriptase domain confirmed that the schistosome element was most closely related to *CsRn1* from the liver fluke *Clonorchis sinensis* [46] and to *kabuki* from the silk moth *Bombyx mori* [47]. We named the schistosome element *Boudicca*, and copies were found throughout the genome of the parasite. In addition, mRNA transcripts of *Boudicca* were present in adult worms, sporocysts, and cercariae. This suggested that *Boudicca* is an active and transcribed element in *S. mansoni* [44]. 

Our laboratories have also identified another new LTR retrotransposon, *Sinbad*, in schistosomes, which belongs to the *Pao/BEL*-like elements. Like *Boudicca*, the full-length *Sinbad* transposon is flanked by LTRs and contains GAG, protease, reverse transcriptase, RNAseH, and integrase motives. It is not as abundant as *Boudicca*, and analyses suggested that there are ~50 copies in the schistosome genome. Transcripts of *Sinbad* could be found in the developmental stages of schistosomes, which, again, suggested that *Sinbad* is actively transcribed. In many organisms, endogenous and exogenous TEs such as *Sleeping Beauty*, *piggyBac*, and *mariner* have been exploited to achieve transfection [48]. It was, therefore, reasonable to assume that schistosome TEs could also be used to achieve not only transient transfection as with biolistic methods but also integration into the genome [49]. The LTRs of *Boudicca* and *Sinbad* were further studied to investigate whether they could function as promotors and, therefore, be used as vehicles for transgene introduction. The analysis revealed that the LTRs contained TATA boxes, polyadenylation signals, and direct inverted repeats. In addition, the LTRs of *Sindbad* were able to drive luciferase expression in both the forward and inverted orientations in HeLa cells [50].

Although these elements held promise for the development of a transfection system for schistosomes, their potential was not realised partly because the development of other techniques superseded the use of mobile genetic elements.

The only success using an exogenous transposable element for transfecting schistosomes was reported by Morales et al. (2007) [51]. The Class II transposon *piggyBac* from the genome of the cabbage looper moth [52] was used to deliver a *piggyBac* donor plasmid that encodes firefly luciferase under the control of schistosome gene promoters into the genome of *S. mansoni* (Figure 2B). Southern hybridization and retrotransposon-anchored PCR analyses showed that the *piggyBac* transposon had integrated into numerous sites within the parasite’s chromosomes [51]. This was the first report to demonstrate that somatic transgenesis is possible in schistosomes. 

### 3.3. RNA Interference

Sequence-specific gene silencing using double-stranded RNA (dsRNA) has been an invaluable tool for investigating gene function. Through the process of RNA interference (RNAi) or RNA silencing, dsRNA pairs with their homologous mRNA targets which prevents gene expression either via mRNA degradation or interference with protein translation (Figure 2C). This method has dominated functional genomics studies in schistosome research for the past 20 years.

Cathepsin B is a key cysteine protease in the digestion of haemoglobin by schistosomes and has also been used as a vaccine target. To confirm a functional role of SmCB1 in parasite survival, Skelly et al. (2003) reported the use of RNAi to inhibit *SmCB1* in adults *in vitro* and confirmed the knockdown with qPCR, immunostaining, and enzyme activity assays [53]; a later report by the same group reported greatly enhanced uptake of dsRNA after using electroporation [54]. Other groups have reported that the suppression of *SmCB1* leads to growth retardation [55] and, further, that SmCB1 knockdown could be achieved using a mouse Moloney virus vector (MMLV) [56]. 

Following the establishment of the RNAi technique in schistosomes, this approach has been used to investigate the function of a wide range of genes in adults, eggs, and schistosomulae. To date, the targeting of over 50 genes in *S. japonicum* and around 80 in *S. mansoni* has been reported, including a number of RNAi screen studies (discussed below). A detailed discussion of these is beyond the scope of this review. The reader is referred to reviews [57,58], and a comprehensive list of studies using RNAi may be found in Supplementary Table S1.

#### RNAi In Vivo

In 2018, Li et al. reported a protocol to achieve RNAi in schistosome life-cycle stages in mammalian hosts. Initially, infected mice were treated with 10 μg of long dsRNA that targets *SjCB1* via intravenous injection. This method was found to induce a knockdown effect of 79% in male worms and 92% in female worms compared with controls, as detected using qPCR [59]. Further optimisation studies showed that the long-term suppression of gene expression could be achieved with 10 μg of dsRNA administered in six doses over 26 days. The effect of suppressing three functional genes (peroxiredoxin, Mago nashi, insulin receptor) could be confirmed with qPCR and morphological changes observed in worms isolated from treated mice. These protocols provided an important advance in facilitating the functional analysis of genes involved in worm development.

The same group went on to investigate the role of *S. japonicum* ferritin genes in maturation. Ferritin proteins store iron and might be expected to play key roles in the processing of the iron-rich blood diet of the worms. The knockdown of three ferritin genes *in vivo* revealed that *SjFer0* affected the growth and development of schistosomulae but did not affect egg production in adults. *SjFer1* and *2* did not affect growth or development [60]. More recently, investigations on the role of the signal peptidase complex (SPC), an essential part of protein translocation machinery, revealed that the *in vivo* knockdown of the *SPC25* gene led to the degeneration of reproductive organs and reduced egg production, reducing damage in the host [61].

### 3.4. Highlights of Findings from RNAi Studies

Collins et al. (2013) identified neoblast-like cells in adult schistosomes, termed proliferating somatic cells (PSCs). These cells were identified in the gut and muscle but not in reproductive organs [62]. PSCs were found to express an ortholog of fibroblast-like growth factor receptor (SmfgfrA). FGF is known in other organisms to play a role in cell proliferation, differentiation, and survival. Using labelling experiments, PSCs were shown to self-renew and differentiate. The inhibition of *SmfgfrA* using RNAi reduced the proliferation of PSCs and cell-cycle-associated gene expression. The findings of this study provide insight into how the parasites survive over a very long period in the host and how they can recover from tissue damage after sublethal doses of praziquantel, for example [63]. 

While most reports have focussed on a single gene or 2–3 genes in a specific pathway, wider-scale RNAi screens are required to identify potential novel drug targets. Initially, Mourão et al. (2009) performed a screen of 32 genes using synthesised dsRNA by transforming miracidia into sporocysts in the presence of dsRNA [64]. Developing larvae were monitored for phenotypic changes, including motility and altered growth, and transcript expression. Of the genes tested, 33% induced a size reduction, but only six of these genes demonstrated a significant and consistent knockdown of the targeted gene; unexpectedly, one gene was not inhibited but rather highly induced. Of the dsRNA treatments that did not result in phenotypes, only some exhibited consistently reduced transcript levels. The variable efficacy of RNAi treatment and the potential for off-target effects have demonstrated the need for optimisation and careful interpretation of data.

Stefanic et al. (2010) investigated selectivity and sensitivity using long dsRNA that targets a set of 11 genes that are known to be located in different tissues [65]. Five of the genes had previously been shown individually to be required for parasite survival. Mechanically transformed schistosomulae were incubated for up to 3 weeks. RNAi was found to be selective, with sensitivity ranging from 40 to 75%, as determined via qPCR, but notably, no obvious phenotypes were observed. 

**Figure 3:**
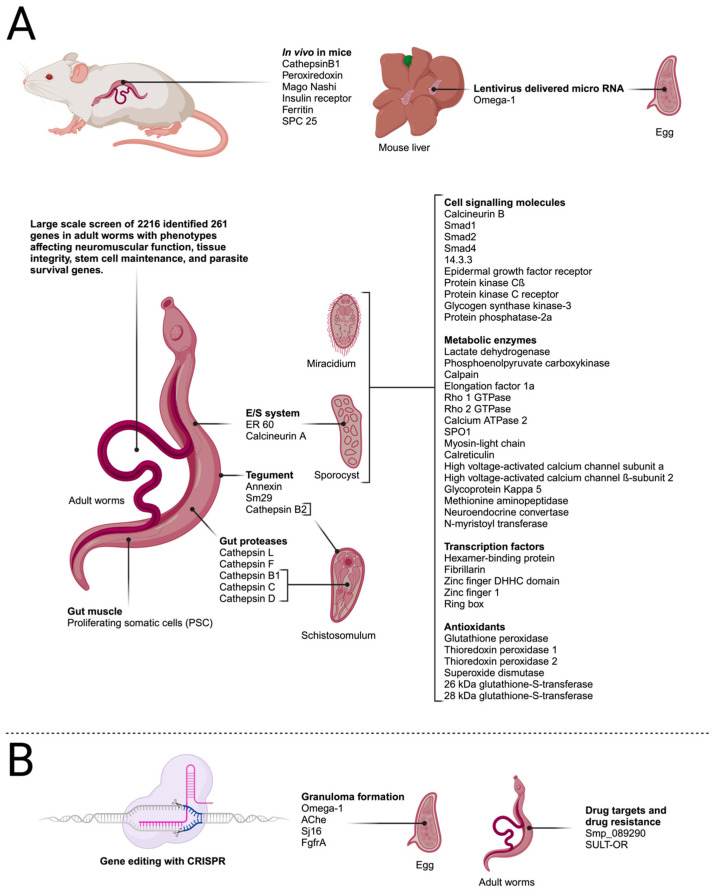
Transfection targets in schistosome life-cycle stages described in the text. (**A**) Genes targeted by RNA interference. (**B**) Genes targeted by gene editing using CRISPR/Cas9. Created with BioRender.com.

A large-scale screen of adult *S. mansoni* targeted 2216 genes by treating adult male and female pairs with dsRNA for 30 days [66]. Substrate attachment (as a proxy of vascular attachment and normal sucker function) and the maintenance of neoblasts and germline stem cells were monitored with ethynyl deoxyuridine (EdU) labelling. The specificity of RNAi targeting was confirmed via sequencing, and the possibility of potential off-target effects was mitigated by testing additional dsRNAs. Using this method, 195 genes were identified that had attachment phenotypes, of which 121 had additional phenotypes. In addition, 66 genes resulted in stem cell maintenance defects in male testes and neoblasts. A screen of existing pharmacological agents identified compounds that could potentially target 49 of the schistosome proteins that exhibited attachment phenotypes. In vitro and *in vivo* testing led to the identification of two compounds that inhibited the proteosome (ubiquitin–proteasome (UPS) component p97), reduced ubiquitinylation *in vitro*, and prevented egg deposition *in vivo* in a mouse model. In addition, two inhibitors of protein kinases were tested and shown to cause deformation in adults and reduced survival *in vivo*. These results illustrate the utility of RNAi not only for refining drug targets but also for confirming the drug’s action. Further, the schistosome UPS and tao and stk25 kinases have been shown to be druggable targets [66]. The studies using RNAi described here show substantial advances in defining new drug and vaccine targets and have permitted genome-wide screens to simultaneously identify additional targets and biochemical pathways for intervention. These studies are summarised in Figure 3.

## 4. Genome Editing Using CRISPR

After the discovery of the clustered regularly interspaced short palindromic repeats/CRISPR-associated nuclease 9 (CRISPR/Cas9) system and developing technology that can use the system for genome editing, it was only a question of time before this technology was also applied in parasitology research and, in the context of this review, schistosome research.

The CRISPR/Cas9 system was developed from an ancient bacterial immune system, which enables prokaryotes to maintain an immune memory after infection with bacteriophages. If the bacteriophage is encountered again, the phage DNA is cut, disabling the invading virus.

The CRISPR/Cas9 system requires three components, a guide RNA (gRNA), the CRISPR-associated (Cas) nuclease, and a protospacer adjacent motif (PAM) site on the complementary strand of the DNA target (Figure 2D). A complex forms between the Cas nuclease and the gRNA, which is used as a search tool to find sequences that are complementary to the gRNA sequence. If a sequence is found, the Cas nuclease binds to the target DNA. The third trigger for executing the cutting of the target DNA is the presence of the PAM site, located downstream of the target, and as a consequence, the Cas will create a double-stranded break in the target DNA. The breaks are subsequently repaired via homology-directed repair (HDR) or non-homologous end joining (NHEJ). Because HDR employs a homologous template that triggers this repair by providing an artificial DNA template, it is the main method of achieving gene replacement or knock-ins. NHJE, on the other hand, is error-prone and leads to mutations that can disable the gene. This method is, therefore, used to generate gene knockout mutations.

### 4.1. Using CRISPR/Cas9 to Understand Mechanisms of Pathogenesis

The first report to describe the application of CRISPR/Cas9 to schistosomes was published by Ittiprasert et al. (2019). The authors investigated the *S. mansoni Omega-1* gene, which is secreted from the eggs of the parasite and is the key protein involved in circumoval granuloma formation in infected hosts. Granulomata are formed as a consequence of the immune response of the host directed against trapped eggs in the tissues, and they are the main cause of pathology in schistosomiasis. The authors used CRISPR-mediated programmed gene editing to further analyse the role of the ribonuclease Omega-1 in the immunopathology of schistosomiasis, which had previously been investigated using an RNAi approach [67,68]. For knockdown studies, CRISPR/Cas9 under the control of the translational elongation factor 1 promotor and *Omega-1*-specific sgRNA driven by the human U6 promotor were delivered into schistosome eggs using a bicistronic lentivirus. In addition, a single-stranded oligodeoxynucleotide was delivered as a template for the HDR of CRIPR/Cas9-induced double-strand breaks. This repair template contained a small transgene that encodes six stop codons to efficiently abrogate the translation of the HDR-repaired *Omega-1* gene locus. The authors were able to demonstrate a 67–83% reduction in the transcript levels of Omega-1 in eggs, with a corresponding reduction in ribonuclease activity. Additionally, T cell responses and cytokine production were investigated, and a mouse lung granuloma model was used to study granuloma formation. The results showed that Th2 cytokines were reduced after exposure to knockdown eggs and that the volume of granulomata in the lungs was reduced 18-fold compared with experiments with wild-type eggs [68].

The formation of granulomas was also investigated by You et al. (2021). In their study, the *S. mansoni* acetylcholinesterase (AChE), which occurs in the eggs of the parasite and in granulomata surrounding the eggs in host tissues, was investigated. Previously, AChE was shown to inhibit the host’s IL-4 responses [69]. In the study by You et al. the authors used two gRNAs that target exon 5, exon 7, or both exons 5 and 7 of the *AChE* gene. Similar to the previously described studies with *Omega-1* [68], Cas9-induced double-strand DNA breaks in the *AChE* gene in worm eggs were repaired by providing repair templates encoding stop codons in all reading frames to efficiently abrogate *AChE* translation [70]. The various knockdown eggs were then used to address questions regarding the immune response to parasite eggs using a mouse granuloma model, where eggs were injected into the lateral tail vein, which led to the formation of granulomata in the lung. When AChE knockdown eggs were injected, the area occupied by the granulomata in the lung was reduced between 4.3- and 10-fold depending on the gRNA that were used for the experiment. In addition, collagen transcription levels in the granulomata were reduced by 46–75%. When cytokine responses were analysed, IL-2, 4, 5, 10, and 13 in the lung and IL-4, 5, 10, and 13 in splenocytes increased five-fold. This result confirms the role of AChE in inhibiting the host’s IL-4 response [69]. When a gRNA that targets exon 5 was used to knockdown AChE, IL-6 and TNFα levels were enhanced seven-fold in splenocytes in comparison with control experiments [68]. Finally, flow cytometry revealed that the numbers of activated CD44^hi^ CD4^+^ cells GATA3^+^, and IL-4^+^ T cells significantly increased. Taken together, these results indicate that blocking the expression of AChE leads to the pronounced shift of the immune response into a Th2 response.

Zhang et al. (2022) also examined granuloma formation. They investigated Sj16, a protein that was identified in *S. japonicum* and is a homolog of Sm16 from *S. mansoni*. Recombinant Sj16 has been shown to have anti-inflammatory properties *in vivo* and *in vitro* [71]. The mechanisms by which this protein modulates the immune response are not yet fully understood. In this study, Sj16 was knocked down using a gRNA that targets exon 4 of the *Sj16* gene. The mouse lung granuloma model was again employed to analyse the effects of the knockdown. Inflammatory responses were more severe after injecting Sj16 knockdown eggs, concurrent with a significant increase in granuloma sizes. An analysis of cytokine secretions in the lung showed that levels of IL-1β, IL-5, IL-6, and IL-10 had also increased [72]. These results suggested that Sj16 downregulates the inflammatory immune response against schistosome eggs, which corroborates earlier results [71].

The most recent publication addressing aspects of granuloma formation targeted the *SmfgfrA* gene of schistosomes, which encodes fibroblast growth factor receptor A. In this investigation, mice that were injected with knockdown eggs exhibited significantly reduced granuloma sizes in addition to a reduction in serum IgE levels. These results indicated that SmfgfrA plays a role in regulating the immune response in schistosome infections.

### 4.2. CRISPR as a Tool for Validating Novel Drug Targets

As mentioned above, praziquantel has been the mainstay of schistosomiasis control for over 50 years. There are, however, concerns about emerging praziquantel resistance [73,74,75], and alternative drugs are urgently needed. No new drugs are currently in clinical trials, and the only alternative to praziquantel is oxamniquine, but it is only active against *S. mansoni* [76].

RNA interference and CRISPR technology now offer the possibility of validating potential drug targets, and the silencing of corresponding genes can be informative for drug target selection.

Hulme et al. (2022) investigated two *S. mansoni* glycosyl hydrolases. These enzymes are responsible for the regulation of glycans on proteins and lipids by removing the terminal α-D-galactose and α-N-acetylgalactosamine residues from glycosylated substrates. In schistosomes, it has been shown that the production of specific glycans is developmentally regulated and that they play a role in the interaction between the parasite and snail hosts and between male and female worms and that some have immunomodulating properties [77,78,79]. An interrogation of the genome sequence of *S. mansoni* indicated that Smp_089290 contains the necessary functional amino acid residues to suggest α-GAL/α-NAGAL activity in the expressed protein [79]. The authors then used CRISPR/Cas9 editing to disrupt the *Smp_089290* gene in adult worms, which resulted in decreased enzyme activity, along with a significant reduction in egg production. The authors concluded that critically important glycosyl hydrolases of the worms could be used as targets for the development of a new class of anti-schistosomal compounds [79].

### 4.3. CRISPR as a Tool for a Transgenesis System

Despite the considerable advances in functional genomics using the CRISPR method, a tractable transgenesis system for schistosomes has yet to be developed. The latest publications that employ the CRISPR system are starting to address the missing links for the development of powerful functional genomics tools for schistosomes.

Sankaranarayanan et al. (2020) investigated the possibility of an oxamniquine-resistant stable transgenic schistosome line. In their study, CRISPR-Cas9 was used to introduce mutations into the sulfotransferase (*SULT-OR*) gene of adult worms. The disruption of the gene is predicted to confer resistance toward the drug oxamniquine. Sequencing analysis revealed that a number of deletions were introduced into the gene after genome editing, which could lead to frame-shift mutations or could cause mRNA degradation via the nonsense-mediated mRNA decay pathway. Despite these encouraging results, the authors were not able to show a knockdown effect at the mRNA level, and it was argued that, possibly, the cells that contain deletions only represent a fraction of adult worm cells expressing SULT-OR [80]. If knockdown efficiencies can be improved in the future, this could lead to the exciting prospect of developing a stable transgenic line of oxamniquine-resistant worms. This would offer the possibility of using oxamniquine resistance as a selectable marker for transfection studies.

Another line of research is trying to improve and adapt the CRISPR system for use in schistosomes. Schistosomes have an AT-rich genome that may not provide the appropriate PAM sequences for Cas9, which requires the NGG motif for efficient gene editing. To address this potential problem, Cas9 gene editing was compared with gene editing with Cas12a, which recognises a TTTV PAM motif. The gene editing efficiencies of Cas9 and Cas12a were compared by targeting the schistosome *Omega-1* gene. Cas9 was less efficient than Cas12a in generating gene knockouts but mediated more precise transgene insertions [81]. Overall, the effect of both nucleases with regard to effects on transcription levels was similar, and using Cas12a did not improve editing efficiencies. Genome editing using the CRISPR system exhibits precision in site-specific gene editing, yet rare off-target effects can occur, and Cas9 occasionally acts on untargeted genomic sites. This may lead to adverse outcomes [82]. 

### 4.4. Genomic Safe Harbour Sites

For other organisms, genomic loci, also called genomic safe harbour sites (GSH), have been identified that can be used for stable and efficient transgene expression without adverse effects on cellular functions after gene editing [83].

Ittiprasert et al. (2023) were able to identify potential GSH sites in *S. mansoni* [84]. By targeting parasite eggs and using a gRNA to target these sites, they were able to introduce an EGFP marker gene via homology-directed repair. Fifteen days after the transfection of parasite eggs, the expression of the fluorescent marker EGFP was detected in the developing larvae in 75% of transfected eggs [84]. The possibility of transfecting eggs without detrimental effects on their biology shows exciting promise for achieving germline transfection and the establishment of transgenic lines of schistosomes.

## 5. Concluding Remarks

This review traces the development of approaches to generate transgenic schistosomes, which has been an essential step in elucidating pathophysiology, developmental biology, and underlying biochemical pathways. As for other major pathogens, transgenesis approaches have now been employed to define and validate intervention targets in the different life-cycle stages of this parasite, which can now be targeted for the control of this devastating and neglected tropical disease.

The initial publications using biolistic methods and transposable elements were able to establish that the insertion of transgenes into schistosomes was possible in principle, but the technical difficulties and the restriction of this method to a few life-cycle stages offered limited opportunities for true functional genomics studies in these parasites. This changed dramatically with the introduction of RNA interference, which enabled reverse genetics by generating null alleles, creating non-functional genes. Some disadvantages of this method are that phenotypes may not be obvious if other redundant genes are present and that knocking down essential genes could lead to lethal phenotypes, making it difficult to investigate the function of these genes. Nonetheless, the application of this technology to schistosomes has led to a wealth of new information about hitherto unknown gene function, and, in addition, RNAi has enabled genome-wide screens and, importantly, the application of *in vivo* RNAi to experimental hosts.

The relatively recent application of genome editing methods using CRISPR technology holds the promise of enabling in-depth investigations of critical pathways in the parasite and finding mechanisms that are important for the host–parasite interplay.

Despite these advances, several hurdles remain, such as establishing stable transgenic lines of schistosomes and, in particular, the lines of the parasite that express an antibiotic resistance gene, which could be used for the easy selection of mutants to generate parasite lines for reverse and forward genetic studies. The principal feasibility of this approach was shown in a report utilising the retroviral-based integration of a transgene that encodes neomycin phosphotransferase into the germline of parasite eggs [85]. Schistosomulae that developed from the eggs were resistant to the antibiotic G418, and a PCR analysis of eggs released from transgenic worms confirmed that the transgene was transmitted to the next generation. However, the integration of the transgene in the genome occurred randomly across all chromosomes, which might lead to an undesired disruption of essential genes. With the ability to target GSH sites in the genome using CRISPR editing, now the potential exists to establish stable antibiotic-resistant lines of schistosomes without affecting essential genes. This would open the way for deriving parasites with gain-of-function or loss-of-function mutations.

A hurdle that might be more difficult to overcome for loss-of-function mutations is the need to create homozygous rather than heterozygous mutants to avoid rescuing the phenotype via the unaffected allele. A potential solution was reported in a human stem cell model, where it was shown that using sense combined with antisense repair templates in HDR leads to a markedly higher frequency of homozygous transgenic mutants [86]. These recent developments should mean that the generation of transgenic/mutant lines will soon become a reality in schistosomes. The availability of mutant parasites will undoubtedly enhance our understanding of critical pathways in the parasite and of mechanisms that are important for host–parasite interplay. This, in turn, will lead to new strategies and methods of combatting and managing schistosomiasis.

## Data Availability

Not applicable.

## Supplementary Materials

The following supporting information can be downloaded at: https://www.mdpi.com/article/10.3390/biology13010048/s1: Table S1: Studies employing RNAi in schistosomes.
